# Comparison of Intra-operative Pressure-Controlled Ventilation and Volume-Controlled Ventilation in Bariatric Surgery: A Prospective Randomized Study

**DOI:** 10.7759/cureus.17567

**Published:** 2021-08-30

**Authors:** Mehmet Salih Sevdi, Serdar Demirgan, Kerem Erkalp, Melahat Karatmanlı Erol, Ali Ozalp, Yuksel Altinel, Aysin Alagol

**Affiliations:** 1 Department of Anesthesiology, Bağcılar Training and Research Hospital, University of Health Sciences, Istanbul, TUR; 2 Department of Anesthesiology, Istanbul University-Cerrahpaşa Institute of Cardiology, Istanbul, TUR; 3 Department of General Surgery, Bağcılar Training and Research Hospital, University of Health Sciences, Istanbul, TUR

**Keywords:** bariatric surgery, general anesthesia, peripheral tissue oxygenation, pressure-controlled ventilation, obesity, volume-controlled ventilation

## Abstract

Background: Mechanical ventilation may be particularly challenging in obese patients undergoing laparoscopic bariatric surgery. The present study aimed to compare the effects of pressure-controlled ventilation (PCV) with those of volume-controlled ventilation (VCV) on peripheral tissue oxygenation (PTO), respiratory function, hemodynamic status, and ventilation-related complications in patients undergoing laparoscopic bariatric surgery.

Methods: A total of 100 patients with obesity who underwent gastric plication or sleeve gastrectomy were recruited for the study, and 60 patients (n=32, in group PCV; n=28, in group VCV) were ultimately enrolled. Data on peri-operative PTO (arterial blood gas [ABG] analysis and tissue oxygen saturation [StO_2_]) and respiratory functions were recorded for each patient, along with post-operative hemodynamic status, fluid intake, urinary output, Numeric Pain Rating Scale (NPRS) score , and complications.

Results: The two groups were similar in pH, partial pressure of oxygen, partial pressure of carbon dioxide, oxygen saturation, and lactate values at baseline, intra-operative and post-operative periods. The peri-operative StO_2_ values were also similar between the two groups at all times. The two groups were identical in terms of preoperative values for respiratory function tests and post-operative hemodynamic status, fluid intake, urinary output, pain scores, and complication rates.

Conclusions: In conclusion, the choice of the mechanical ventilation mode did not appear to influence oxygen delivery, respiratory function, hemodynamic status, post-operative pain, or ventilation-related complications in obese patients undergoing laparoscopic bariatric surgery.

## Introduction

The effect of obesity on lung mechanics is associated with excessive fatty tissue, common respiratory comorbidities, and the altered pharmacokinetics of drugs [1]. Given the steady global increase in the prevalence of obese patients undergoing surgery, whether bariatric or non-bariatric, the provision of adequate ventilation to these patients is becoming particularly challenging as a consequence of such patients’ obstructive and restrictive pulmonary deficits and abnormal ventilatory mechanics during pneumoperitoneum [1-5]. The inverse relationship between the arterial partial pressure of oxygen (PaO_2_) and body mass index (BMI) is documented for anesthetized patients, along with the increased risk of peri-operative complications such as hypoxemia, hypercapnia, atelectasis, and wound infection in obese patients undergoing surgery [6-8].

Although different intra-operative ventilation strategies have been evaluated in several studies, the ideal ventilation strategy for obese patients undergoing surgery has yet to be defined [9,10]. Pressure-controlled ventilation (PCV) decelerates the inspiratory flow. It allows a high initial flow rate, faster alveolar inflation, and, consequently, a more homogeneous distribution of the delivered gas mixture and better ventilation-perfusion matching besides the reduced risk of volutraumas and barotraumas [1,11,12]. Although improved oxygenation through the use of intra-operative PCV rather than volume-controlled ventilation (VCV) has been reported in some studies, there is as yet no data suggesting the benefits of intra-operative PCV or VCV in terms of clinical outcomes among obese patients [9,13-16].

Given the lack of clear evidence on the most effective intra-operative ventilation strategy in this specific patient population, the present study has been designed to compare the use of intra-operative PCV and VCV in obese patients undergoing laparoscopic bariatric surgery in terms of peripheral tissue oxygenation (PTO), respiratory functions, and hemodynamic status and post-operative mechanical complications [9].

## Materials and methods

Study population

**Figure 1:**
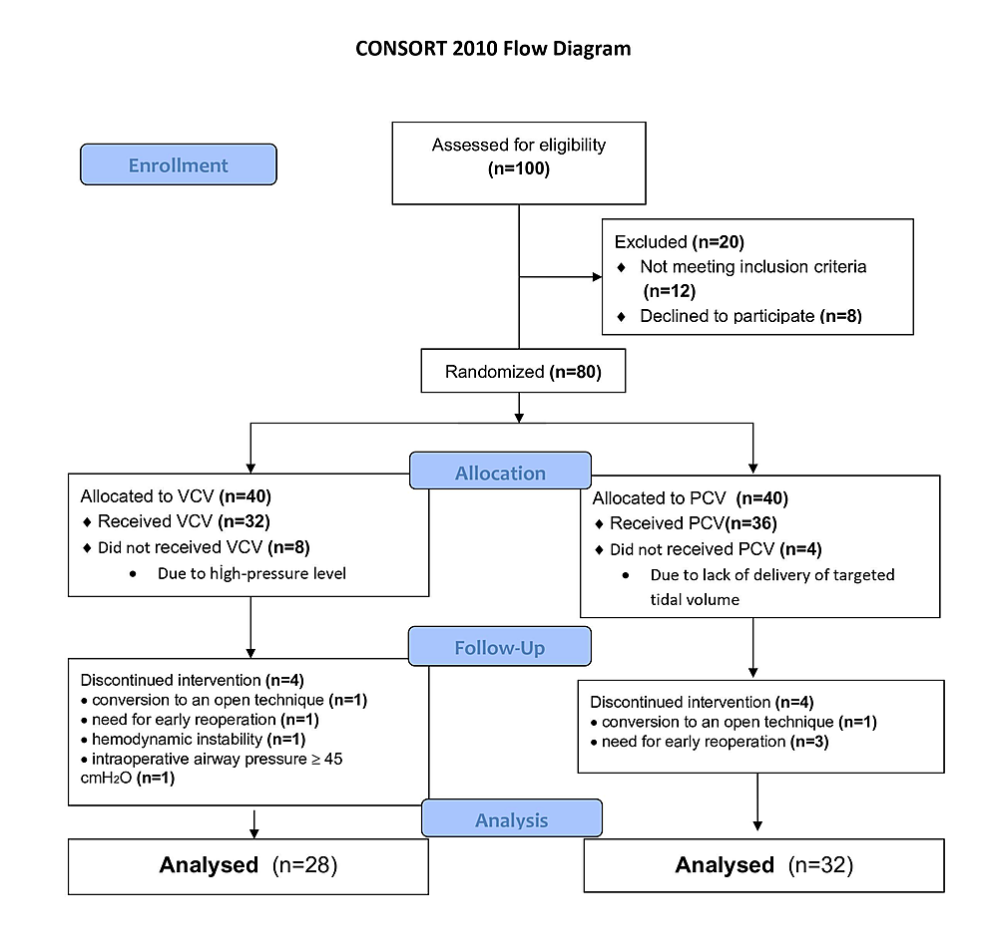
Flow diagram for patient enrollment CONSORT, CONsolidated Standards Of Reporting Trials; VCV, volume-controlled ventilation; PCV, pressure-controlled ventilation

As illustrated in the CONsolidated Standards Of Reporting Trials (CONSORT) flow diagram, after 100 patients were assessed for eligibility, 80 obese patients scheduled for laparoscopic gastric plication or sleeve gastrectomy were included in the study after the exclusion of 20 patients who did not meet the inclusion criteria (n=12) or who declined to participate in the survey (n=8). The patients were assigned randomly to the group VCV (n=40) or group PCV (n=40), using a set of computer-generated random numbers concealed in sequentially numbered, opaque, sealed envelopes. The 60 patients (n=32, in group PCV; n=28, in group VCV) were ultimately enrolled in the study (Figure 1).

The inclusion criteria were age under 18 years, a BMI of 30-45 kg/m^2^, and an American Society of Anesthesiologists (ASA) physical status II-III, which is updated as the ASA’s Physical Status Classification System to include obesity as a metric to consider when determining a patient’s physical status. Also, the patients who had approval from the endocrine multidisciplinary team were included in the study. Exclusion criteria were lack of or inability to give written informed consent, hepatomegaly detected upon a preoperative assessment, jugular vein distension, tibial edema, and pulmonary rales, negative modified Allen’s test, anemia (hemoglobin concentration <10 g/dl), a history of cardiac disease, obstructive or restrictive lung disease, previous thoracic surgery, Raynaud’s disease, a peripheral arterial disease such as thromboangiitis obliterans, peri-operative complications (i.e., hemodynamic instability, need for peri-operative inotropic drugs or pneumothorax), intra-operative airway pressure of ≥45 cmH_2_O, conversion to an open technique or need for early reoperation.

Written informed consent was obtained from each participant following a detailed explanation of the aim and protocol of the current study, which was conducted following the ethical principles stated in the “Declaration of Helsinki” and approved by the Bağcılar Training and Research Hospital Ethics Committee (date of approval: February 1, 2013; reference number: 228).

Study parameters

The patients’ demographics (age, gender, BMI, ASA score, duration of anesthesia, and surgery [minute]) were reported. The mean arterial pressure (MAP), heart rate (HR), end-tidal carbon dioxide (etCO_2_), and tissue oxygen saturation (StO_2_) values of the two groups were compared at 15, 30, 45, and 60 minutes of pneumoperitoneum. Peri-operative (baseline), intra-operative (30 and 60 minutes of pneumoperitoneum) and post-operative (1, 4, 8, 12, and 24 hours) arterial blood gas (ABG) parameters (pH, partial pressure of oxygen [PaO_2_, mmHg], partial pressure of carbon dioxide [PaCO_2_, mmHg], oxygen saturation [SaO_2_, %] and lactate [mmol/l]) and StO_2_ were evaluated in study groups, as were preoperative respiratory functions (forced vital capacity [FVC], forced expiratory volume in the first second [FEV1], FEV1/FVC) and post-operative MAP, heart rate (HR), oxygen saturation (SpO_2_), body core temperature (°C), fluid intake (ml/h), urinary output (ml/h), Numeric Pain Rating Scale (NPRS) scores, and post-operative complications such as bronchospasm, desaturation, and hypercapnia.

Procedures

The patients fasted for eight hours before the induction of anesthesia and were administered premedication (3 mg midazolam intravenously, 40 mg enoxaparin subcutaneously, 50 mg ranitidine, and 10 mg metoclopramide intravenously). Routine monitoring of the electrocardiogram, non-invasive blood pressure (NIBP), and SpO_2_ was performed, and intravenous catheterization was administrated for intravenous access. Baseline HR, MAP, SpO_2_, and StO_2_ (InSpectra StO2 Spot Check 300; Hutchinson Technology, Hutchinson, MN, USA) were recorded before the induction of anesthesia. Radial artery catheterization and invasive blood pressure monitoring were established on the non-dominant side, and samples were taken for baseline blood gas analysis (ABL 835; Radiometer, Brønshøj, Denmark).

Ringers’ lactate (10 ml/kg) was administered intravenously over 15 minutes before the induction of anesthesia. Anesthesia was applied based on the ideal body weight (IBW) by administering oxygen by facemask (80% O_2_) for three to five minutes, followed by propofol 2 mg/kg and fentanyl 2 µg/kg. Rocuronium (0.6 mg/kg) was given intravenously based on IBW before endotracheal intubation. The patients were placed in a 25° head-up position, and balanced anesthesia was maintained with sevoflurane 1%-2% in a mixture of oxygen and air (fraction of inspired oxygen [FiO_2_] 0.5 at a flow rate of 2 l/min in both groups [Datex Ohmeda S/5 Avenue; GE Healthcare, Madison, WI, USA]). The patients had mechanically ventilated an inspiratory time/expiratory time ratio of 1:2, positive end-expiratory pressure (PEEP) of 5 cm H_2_O, and a tidal volume of 8-10 ml/kg adjusted according to the ideal weight calculation (50 + 0.91 × [height, in cm - 152.4], for men; or 45.5 + 0.91 × [height, in cm - 152.4], for women) [14]. In group PCV, the patients were ventilated at positive inspiratory pressure (PIP), adjusted to the same tidal volume as the patients in group VCV. The respiratory frequency was adjusted to maintain an end-tidal pressure of carbon dioxide (EtCO_2_) at 35-45 mmHg in both groups. The SpO_2_, StO_2_, MAP, HR, and EtCO_2_ were recorded at baseline (except for EtCO_2_) and at 15, 30, 45, and 60 minutes of pneumoperitoneum. The StO_2_ probe was positioned so that it covered the thenar eminence of the dominant hand. Carbon dioxide was insufflated into the peritoneal cavity until the intraabdominal pressure reached 12 mmHg, which was maintained throughout the procedure. Anesthesia was discontinued at the end of the surgical procedure, and metoclopramide 10 mg, ondansetron 4 mg, paracetamol 1 g, and morphine 0.1 mg/kg were administered intravenously before extubation. No recruitment maneuvers were performed during anesthesia. All operations were performed by surgeons with over 10 years of experience.

After surgery, all patients were transferred to the intensive care unit (ICU), where MAP, HR, SpO_2_, and StO_2_ were monitored. All of the patients were administered supplemental oxygen via a facemask at a flow rate of 4 l/min. An ABG analysis was made at the 1, 4, 8, 12, and 24 hours after ICU admission. Post-operative pain was treated with tramadol through a patient-controlled analgesia system. A bolus of 5 mg morphine and 1 mg paracetamol was given intravenously for rescue analgesia. Fluid management, supplemental oxygen administration, and analgesia were managed by the ICU team. Post-operative respiratory function tests were performed 24 hours after surgery.

Statistical analysis

The statistical analysis of the data was based on means, standard deviations, ranges, medians, ratios, and frequencies. The distribution of variables was controlled using the Kolmogorov-Smirnov test. The independent variables were analysed using independent samples t-tests, Mann-Whitney U test, and chi-square test. SPSS 21.0 software (IBM Corp, Armonk, NY, USA) was used to analyse the data.

## Results

A total of 100 patients were assessed for eligibility in the preoperative period, after which 40 of the patients were excluded, and 60 patients were ultimately enrolled in the study (Figure 1). There were no significant differences in the demographic or clinical characteristics of the patients in groups VCV and PCV (Table 1).

**Table 1:** Demographic and clinical characteristics of the patients Independent samples t-test, Mann-Whitney U test, and chi-square test were used where appropriate. Data are presented as means ± SDs and number of the patients. VCV, volume-controlled ventilation; PCV, pressure-controlled ventilation; ASA, American Society of Anesthesiologists; BMI, body mass index

	Group VCV (n=28)	Group PCV (n=32)	p
Age, years (mean ± SD)	36.5 ± 0.51	37.82 ± 0.76	0.869
Gender (male/female)	15/13	17/15	1.000
BMI (kg/m^2^)	43.5 ± 3.6	43.1 ± 3.6	0.806
Preoperative hemoglobin concentration (mg/dl)	13.8 ± 1.23	13.6 ± 2.21	0.725
Duration of anesthesia (min)	90.95 ± 21.45	92.95 ± 19.35	0.763
Duration of surgery (min)	62.0 ± 2.3	61.6 ± 2.1	0.520

The two groups were similar in pH, PaO_2_, PaCO_2_, SaO_2_, and lactate values at baseline, intra-operative 30 and 60 minutes, and post-operative 1, 4, 8, 12, and 24 hours (Table 2). The peri-operative StO_2_ values were also similar between the two groups at all time points (Table 3). During the surgery, MAP and HR did not reveal significant differences between the groups at any time point (p>0.05).

**Table 2:** Perioperative (baseline, intra-operative and postoperative) arterial blood gas values of the patients p values were based on the Mann-Whitney U test. Data are presented as means ± SDs. VCV, volume-controlled ventilation; PCV, pressure-controlled ventilation; PaCO_2_, partial pressure of carbon dioxide, PaO_2_, partial pressure of oxygen, SaO_2_, oxygen saturation

	Group VCV (n=28)	Group PCV (n=32)	p
	Mean ± SD	Mean ± SD	
pH			
Baseline	7.43 ± 0.03	7.41 ± 0.04	0.122
Intra-operative			
30^th ^min	7.39 ± 0.03	7.37 ± 0.04	0.318
60^th^ min	7.37 ± 0.03	7.37 ± 0.05	0.864
Postoperative			
1^st ^hour	7.38 ± 0.03	7.38 ± 0.03	0.982
4^th ^hour	7.38 ± 0.03	7.38 ± 0.03	0.764
8^th ^hour	7.40 ± 0.02	7.39 ± 0.04	0.352
12^th ^hour	7.40 ± 0.03	7.40 ± 0.04	0.988
24^th ^hour	7.40 ± 0.03	7.41 ± 0.02	0.342
PaO_2_(mmHg)			
Baseline	120.8 ± 35.5	116.7 ± 40.5	0.482
Intra-operative			
30^th^ min	142.0 ± 38.2	125.4 ± 37.2	0.078
60^th^ min	144.7 ± 52.4	126.8 ± 36.2	0.160
Postoperative			
1^st^ hour	123.6 ± 33.1	118.6 ± 38.5	0.351
4^th^ hour	115.9 ± 25.4	108.6 ± 29.1	0.122
8^th^ hour	108.9 ± 25.4	103.5 ± 25.7	0.367
12^th^ hour	108.0 ± 24.9	99.0 ± 22.6	0.237
24^th^ hour	111.1 ± 17.9	102.6 ± 15.6	0.151
PaCO_2 _(mmHg)			
Baseline	36.0 ± 2.7	37.4 ± 4.4	0.198
Intra-operative			
30^th^ min	36.7 ± 3.4	39.1 ± 4.5	0.052
60^th^ min	38.2 ± 3.6	39.1 ± 4.0	0.221
Postoperative			
1^st ^hour	38.6 ± 3.3	38.8 ± 4.2	0.499
4^th^ hour	38.5 ± 4.6	39.0 ± 4.0	0.414
8^th^ hour	37.0± 3.3	38.2 ± 4.7	0.249
12^th^ hour	38.6 ± 3.7	38.2 ± 4.7	0.651
24^th^ hour	37.6± 2.9	38.8 ± 3.9	0.275
SaO_2 _(%)			
Baseline	97.3± 1.6	97.5 ± 2.0	0.231
Intra-operative			
30^th^ min	98.3 ± 1.2	97.7 ± 2.0	0.392
60^th^ min	97.7 ± 1.8	97.4 ± 1.5	0.271
Postoperative			
1^st ^hour	98.0 ± 1.5	97.3 ± 2.0	0.199
4^th^ hour	97.9 ± 1.5	97.4 ± 1.6	0.224
8^th^ hour	97.5 ± 1.6	97.1 ± 1.5	0.346
12^th^ hour	97.2 ± 1.6	96.7 ± 2.4	0.566
24^th^ hour	97.5 ± 1.2	97.1 ± 1.6	0.328
Lactate(mmol/l)			
Baseline	0.9 ± 0.4	1.1 ± 0.6	0.191
Intra-operative			
30^th^ min	1.1 ± 0.4	1.1 ± 0.9	0.212
60^th^ min	1.1 ± 0.6	1.1 ± 0.9	0.275
Postoperative			
1^st ^hour	1.2 ± 1.0	1.2 ± 0.8	0.402
4^th^ hour	1.0 ± 0.8	1.3 ± 0.6	0.155
8^th^ hour	1.0 ± 0.9	1.2 ± 0.6	0.841
12^th^ hour	1.1 ± 0.6	1.0 ± 0.4	0.401
24^th ^hour	1.0 ± 0.6	1.0 ± 0.5	0.970

**Table 3:** Baseline, intra-operative and postoperative tissue oxygen saturation of the patients undergoing laparoscopic bariatric surgery p values were based on the independent samples t-test. Data are presented as means ± SDs. VCV, volume-controlled ventilation; PCV, pressure-controlled ventilation

	Group VCV (n=28)	Group PCV (n=32)	p
	Mean ± SD	Mean ± SD	
Baseline	81.0 ± 7.2	81.2 ± 6.5	0.882
Intra-operative			
15^th^ min	83.6 ± 6.7	85.6 ± 4.8	0.313
30^th ^min	83.8 ± 7.2	84.0 ± 4.4	0.568
45^th^ min	83.4 ± 6.7	84.6 ± 4.4	0.689
60^th^ min	83.5 ± 6.6	84.4 ± 5.0	0.929
Postoperative			
1^st^ hour	84.5 ± 3.9	83.5 ± 4.5	0.536
4^th^ hour	83.3 ± 5.1	81.9 ± 6.3	0.481
8^th^ hour	83.9 ± 6.1	83.6 ± 6.3	0.953
12^th^ hour	82.5 ± 6.6	82.6 ± 5.6	0.994
24^th^ hour	83.0 ± 6.2	83.1 ± 5.1	0.998

In the post-operative period, groups VCV and PCV were similar in terms of MAP, HR, SpO_2_, body core temperature, fluid intake, urinary output, and NPRS scores at any time point (Table 4). The preoperative respiratory function test values did not differ significantly between the groups (Table 5). The values of respiratory function tests that were administrated 24 hours after surgery were also similar between groups. Groups VCV and PCV had a similar rate of complications, including bronchospasm, desaturation and hypercapnia (p=0.705, p=0.465, p=0.633, respectively). There was a significant positive correlation between post-operative StO_2_ and PaO_2_ levels at all time points, including at 1 hour (r=0.560, p=0.01), 4 hours (r=0.548, p=0.002), 8 hours (r=0.578, p=0.02), 12 hours (r=0.600, p=0.01) and 24 hours (r=0.563, p=0.002).

**Table 4:** Postoperative first 24-hour characteristics of the patients undergoing laparoscopic bariatric surgery p values were based on the independent samples t-test. Data are presented as means ± SDs. VCV, volume-controlled ventilation; PCV, pressure-controlled ventilation; MAP, mean arterial pressure; HR, heart rate; SpO_2_, oxygen saturation; NPRS, Numeric Pain Rating Scale

		Group VCV (n=28)	Group PCV (n=32)	p
		Mean ± SD	Mean ± SD	
HR	1^st^ hour	81.5 ± 10.4	81.6 ± 10.2	0.935
	4^th^ hour	82.3 ± 9.9	84.6 ± 10.2	0.420
	8^th^ hour	85.1 ± 11.3	86.1 ± 9.8	0.620
	12^th^ hour	83.5 ± 8.0	84. 9 ± 11.7	0.667
	24^th^ hour	82.1 ± 8.2	85.2. ± 11.6	0.402
MAP	1^st ^hour	104.9 ± 19.5	107.8 ± 14.8	0.270
	4^th^ hour	101.1 ± 17.9	102.3 ± 11.6	0.314
	8^th^ hour	102.2 ± 16.7	101.2 ± 11.5	0.756
	12^th^ hour	101.3 ± 16.5	97.3. ± 12.4	0.579
	24^th^ hour	96.3 ± 10.1	93.9 ± 9.2	0.309
SpO_2_	1^st^ hour	98.0 ± 1.5	97.5 ± 1.9	0.270
	4^th^ hour	97.3 ± 2.0	97.3 ± 1.9	0.733
	8^th^ hour	97.3 ± 1.5	96.9 ± 2.1	0.520
	12^th ^hour	97.1 ± 1.5	97.0 ± 2.0	0.898
	24^th ^hour	97.4 ± 1.0	97.2 ± 1.3	0.818
Body core temperature	1^st ^hour	36.0 ± 0.2	36.1 ± 0.5	0.408
	4^th^ hour	36.5 ± 0.4	36.6 ± 1.1	0.790
	8^th^ hour	36.9 ± 0.3	37.0 ± 0.2	0.172
	12^th^ hour	37.4 ± 0.4	37.2 ± 0.2	0.057
	24^th^ hour	37.3 ± 1.1	37.4 ± 0.3	0.696
Fluid intake (ml/hour)	1^st^ hour	830.5 ± 162.36	785.42 ± 171.3	0.395
	4^th^ hour	868.5 ± 137.5	874.4± 132.5	0.904
	8^th^ hour	868.0 ± 146.7	874.3 ± 141.7	0.896
	12^th^ hour	850.5 ± 150.7	856.5 ± 145.7	0.895
	24^th^ hour	843.5 ± 166.5	849.5 ± 161.5	0.880
Urinary output (ml/hour)	1^st ^hour	126.25 ± 36.01	124.35 ± 34.05	0.865
	4^th^ hour	123.75 ± 36.45	121.65 ± 34.55	0.853
	8^th ^hour	125.00 ± 35.76	123.00 ± 33.46	0.854
	12^th^ hour	125.75 ± 35.58	123.55 ± 33.36	0.840
	24^th^ hour	128.50 ± 35.72	126.50 ± 33.41	0.853
NPRS score	1^st^ hour	2.70 ± 1.03	2.65 ± 1.20	0.885
	4^th^ hour	2.45 ± 0.82	2.50 ± 0.89	0.854
	8^th^ hour	2.15 ± 0.74	1.90 ± 0.24	0.152
	12^th^ hour	1.80 ± 0.76	1.65 ± 0.50	0.460
	24^th^ hour	1.55 ± 1.20	1.40 ± 1.36	0.715

**Table 5:** Intra-operative EtCO2 and preoperative respiratory function test values of the patients p values were based on the independent samples t-test. Data are presented as means ± SDs. VCV, volume-controlled ventilation; PCV, pressure-controlled ventilation; EtCO_2_, end-tidal carbon dioxide; FEV1, forced expiratory volume in the first second; FVC, forced vital capacity

	Group VCV (n=28)	Group PCV (n=32)	p
	Mean ± SD	Mean ± SD	
Intra-operative EtCO_2_ (mmHg)			
15^th^ min	35.8 ± 2.7	35.6 ± 3.6	0.799
30^th ^min	35.2 ± 2.1	36.9 ± 4.5	0.155
45^th ^min	35.8 ± 2.3	37.4 ± 4.4	0.222
60^th ^min	35.6 ± 1.7	36.8 ± 4.1	0.554
FVC (%)	89.0 ± 10.0	88.8 ± 15.9	0.420
FEV1 (%)	95.2 ± 10.0	93.1 ± 14.8	0.201
FEV1/FVC (%)	110.2 ± 8.4	108.1 ± 7.6	0.264

## Discussion

Our findings revealed no significant difference between the groups in terms of peri-operative arterial blood gas and acid-base status, respiratory functions, post-operative hemodynamic status, fluid intake, urinary output, pain scores, and the rate of complications. It is also demonstrated that the choice of mechanical ventilation modes did not affect oxygen saturation in microcirculation (StO_2_) in the patients undergoing bariatric surgery.

Data from randomized trials involving obese patients operated under general anesthesia yielded insufficient evidence for any mechanical ventilation modes to be considered an optimum approach [9,13,16]. Accordingly, data from a systematic review of four randomized controlled trials including 100 obese surgical patients comparing PCV with VCV revealed no evidence of any difference between PCV and VCV regarding PaO_2_/FiO_2_ ratio, tidal volume, or MAP [9]. A meta-analysis of eight randomized controlled trials, including 428 participants, analysing the hemodynamic and respiratory effects of PCV and VCV during laparoscopic surgery revealed similarities in the two ventilation modes in hemodynamic parameters. However, mildly better respiratory functions were reported among patients who underwent PCV [17]. Our findings also reveal similar efficacy with VCV and PCV regarding peri-operative tissue oxygenation, hemodynamic status, and MAP in obese patients. The present study also identified no superiority of either ventilation mode regarding peri-operative arterial blood gas values and acid-base status or in post-operative respiratory function, renal function, pain, and post-operative complications.

Traditional techniques, such as blood gas analysis and venous oximetry, are accurate but invasive. At the same time, transcutaneous near-infrared spectroscopy (TNIRS) is a non-invasive, easily applied technique that has shown promising results in the measurement of PTO in routine practice [18]. TNIRS allows direct measurement of oxygen saturation (StO_2_) in microcirculation, where oxygen is exchanged with tissues. Hence, it could be used for the evaluation of regional, maybe even systemic perfusion. The technique identifies perfusion deficits more promptly than conventional metabolic indices, such as serum lactate concentration and base deficit [19-20]. In our analysis, the measurement of oxygenation based on either ABG analysis or TNIRS revealed no significant difference between ventilation modes. This situation indicates that both arterial and tissue oxygenation is not affected by mechanical ventilation strategies in obese patients undergoing laparoscopic bariatric surgery. Even when the patients’ hemodynamic parameters (i.e., blood pressure, heart rate, and consciousness level) were satisfactory, PTO was reported to facilitate the early detection of a developing critical situation [21-23]. As such, measuring post-operative PTO with TNIRS may be beneficial in obese patients. The impaired subcutaneous oxygenation associated with obesity is considered a significant risk factor for surgical site infection [24]. In contrast, wound infection and tissue hypoxia are reported to be expected in obese patients undergoing surgery, and it is reported that supplemental oxygen only slightly increases PTO [25].

There are various factors with the potential to affect PTO, such as the maintenance of normothermia and normovolemia, adequate pain treatment, mode of anesthesia, and type of surgery [26-27]. Furthermore, PTO has also been reported to decrease during the induction of general anesthesia due to peripheral vasodilatation [28]. In the present study, no impairment of PTO was detected after anesthesia induction, which may be explained by the volume replacement strategy of 10 ml/kg crystalloid and preoxygenation in the preoperative period. Moreover, no significant difference was noted in PTO during the pneumoperitoneum or in the reverse Trendelenburg position. Mild hypercapnia (i.e., an end-tidal PaCO_2_ of 50 mmHg), frequently observed during laparoscopic surgery, has been suggested to improve PTO in morbidly obese surgical patients [29].

Our study has focused on the influence of peri-operative ventilation techniques on post-operative PTO to establish which of the VCV or PCV methods is superior, concluding that the type of ventilation mode does not seem to be a determining factor in improved tissue oxygenation in obese patients undergoing surgery. Accordingly, our findings suggest that the ideal intra-operative ventilation strategy in obese adult patients remains obscure. So the strategy selection should be based on the unique characteristics of the case for the achievement of appropriate lung-protective ventilation and the prevention of both volutrauma/barotrauma and hypoventilation [1].

Certain limitations of this study should be considered. Firstly, the relatively small sample size may prevent statistical significance to be achieved concerning the differences between the two ventilation modes, and may also preclude extensive causal conclusions from being drawn. Secondly, the lack of the groups of morbid obese or non-obese patients limits the full elucidation of the role of mechanical ventilation in obese patients. Thirdly, ventilation was assessed based on both ABG analysis and PTO, but given that similar previous clinical trials have failed to identify a difference between the two ventilation methods (PCV and VCV) through clinical assessment and an ABG analysis, the use of a more sensitive method of assessment for the effect of ventilation modes on tissue perfusion (such as sublingual microcirculation) would have extended the knowledge garnered in the present study.

## Conclusions

In conclusion, our findings related to a cohort of obese patients undergoing laparoscopic bariatric surgery, randomly assigned to receive VCV or PCV intra-operatively, revealed no effect of the peri-operative mechanical ventilation mode on oxygen delivery, respiratory function, hemodynamic status, post-operative pain, and ventilation-related complications. Further studies are needed to investigate the effect of different mechanical ventilation modes on tissue perfusion in patients undergoing bariatric surgery.
